# Impact of Early Stages of the COVID-19 Pandemic on Sexually Transmitted Infection Screening Claims Among Adolescent Females in a Pediatric Accountable Care Organization in Ohio, United States

**DOI:** 10.7759/cureus.32070

**Published:** 2022-11-30

**Authors:** Laura Hart, Fareeda W Haamid, Cynthia Holland-Hall, Andrea E Bonny

**Affiliations:** 1 Internal Medicine/Pediatrics, Nationwide Children’s Hospital, Columbus, USA; 2 Adolescent Medicine, Nationwide Children’s Hospital, Columbus, USA

**Keywords:** accountable care organization, covid-19, adolescent, gonorrhea screening, chlamydia screening

## Abstract

Background

Testing for sexually transmitted infections (STIs) decreased during the early months of the coronavirus disease 2019 (COVID-19) pandemic. Less is known about the extent to which screening of asymptomatic adolescents for STIs was specifically affected. Our aim was to describe the impacts of early stages of the COVID-19 pandemic on asymptomatic STI screening and overall STI testing among adolescent females aged 13 to 19. We hypothesized that screening would decrease more than overall testing.

Methods

We evaluated claims data from a pediatric accountable care organization responsible for approximately 40,000 adolescent females. We assessed rates of asymptomatic screening and overall testing for chlamydia and gonorrhea in this population, comparing the early pandemic to pre-pandemic levels.

Results

Both STI screening and overall STI testing were found to be significantly decreased during the early period of the COVID-19 pandemic compared to pre-pandemic levels. The proportion of tests billed as screening was 70% of tests for April to August 2020 (early pandemic), compared to 67% for October 2019 to February 2020 and 64% for April to August 2019, contrary to our hypothesis.

Conclusion

Asymptomatic screening represented a similar proportion of STI testing among this population of adolescent females during the early COVID-19 pandemic compared to pre-pandemic testing. More work is needed to understand how asymptomatic screening was proportionally maintained despite COVID-19 pandemic restrictions.

## Introduction

Centers for Disease Control and Prevention and the United States Preventive Services Task Force recommend screening for chlamydia and gonorrhea in asymptomatic sexually active adolescent females [1,2]. During the early stages of the coronavirus disease 2019 (COVID-19) pandemic, many primary care offices cancelled or delayed health maintenance appointments. Some appointments that could not be delayed were conducted via telehealth, limiting the opportunity for laboratory testing, including chlamydia and gonorrhea screening.

Studies have shown that, in the early phases of the COVID-19 pandemic, fewer chlamydia and gonorrhea tests were done [3-6]. Additionally, there was an increase in the test positivity rate, suggesting that screening was disproportionately affected compared to symptomatic testing for sexually transmitted infections (STIs) [4-6]. However, these studies did not specifically look at trends in screening (i.e., asymptomatic) testing versus symptomatic testing.

To address this gap, we conducted a retrospective study among adolescent females aged 13 to 19 to evaluate differences in overall chlamydia and gonorrhea testing (i.e., total tests completed) and screening (i.e., testing performed on asymptomatic patients) before and during the early stages of the COVID-19 pandemic. We hypothesized that screening tests would constitute a smaller proportion of total tests completed during the early stages of the pandemic compared to earlier time periods.

## Materials and methods

We queried administrative claims data from Partners For Kids (PFK), a pediatric accountable care organization affiliated with Nationwide Children’s Hospital, for this study. PFK assumes medical and financial responsibility for the care of over 300,000 pediatric Medicaid beneficiaries throughout central and southeast Ohio, including about 40,000 adolescent females. As part of its arrangement with Ohio Medicaid, PFK pays for services rendered by any Medicaid provider and its claims data reflects all claims billed through Medicaid for the covered beneficiaries. As such, we had access to all claims for STI testing and STI screening for PFK participants for the time periods of interest that were billed to insurance, regardless of the clinical site in which testing occurred.

Ohio initiated restrictions aimed at limiting COVID-19 spread starting in mid-March 2020. School closures were initiated on March 14, 2020. Closures of non-essential businesses occurred over the next few weeks [7]. Restrictions were eased starting May 2020, with limitations on indoor capacity and masking requirements in many circumstances. Schools were a mix of in-person and online schooling in the fall of 2020 to limit the number of students in each classroom. We chose to examine the impact of the 2020 restrictions on STI screening compared to testing, since this time period was associated with steepest declines in testing overall. This study was approved by the Nationwide Children’s Hospital Institutional Review Board.

STI testing definitions

Our time period of interest was April to August 2020. These months represented the time between March 2020, when many healthcare institutions and policies in Ohio were in flux due to rapidly changing coronavirus dynamics and September 2020, when limited supplies of STI testing equipment led to changes in how providers addressed routine screening [8]. We selected two comparator intervals to account for seasonal variation by including the same months of the prior year (April to August 2019) and the months immediately prior to COVID-19 restrictions (October 2019 to February 2020).

We identified all chlamydia and/or gonorrhea tests performed in females 13-19 years old during the intervals of interest based on Current Procedural Terminology (CPT) codes (87490, 87491, 87800, 87810, 87590, 87591, 87850), as well as indications for testing as determined by the International Classification of Diseases, 10th Revision (ICD-10) codes associated with each claim. Screening was defined as chlamydia and/or gonorrhea testing done with a screening or health maintenance visit code as the primary diagnosis (ICD codes Z00.00, Z00.129, Z01.419, Z11.3, Z11.8, Z72.51, Z72.53, Z72.89). We excluded testing done during pregnancy.

Analysis

Descriptive statistics were calculated for the number of chlamydia and gonorrhea tests performed overall, number of chlamydia and gonorrhea screening tests conducted, number of individuals tested, and number of individuals screened, both as raw numbers and as total per 1000 member-months to standardize across the time periods of interest.

To assess for differences in screening versus overall testing, we determined the proportion of tests billed as screening tests for each time period and compared these proportions. Chi-square tests and z-tests for differences of proportions were used as appropriate to assess for statistical significance. Analyses were done in Excel (version 2008; Microsoft, Redmond, WA).

## Results

**Table 1:** Chlamydia and gonorrhea testing and screening among adolescent females by time period ^a^Total member-months for each time period are as follows: for April to August 2020, 229,591 member-months; for October 2019 to February 2020, 216,742 member-months; for April to August 2019, 219,691 member-months ^b^Calculated with chi-square testing or z-test for differences in proportions as appropriate ^c^p-value for the z-test comparing the early pandemic to the immediate pre-pandemic time period ^d^p-value for the z-test comparing the early pandemic to the one year before the pandemic time period

Time period	Early pandemic (April to August 2020)	Immediate pre-pandemic (October 2019 to February 2020)	One year pre-pandemic (April to August 2019)	p-value^b^
Number of tests (total)	4206	5348	5648	< .001
Number of tests (total)/1000 member-months^a^	18.32	24.67	25.71	< .001
Number of screening tests	2964	3603	3746	< .001
Number of screening tests/1000 member-months^a^	12.91	16.62	17.05	< .001
Number of individuals tested	1935	2399	2551	< .001
Number of individuals screened	1446	1724	1801	< .001
Proportion of tests done as screening (%)	70	67	64	<.01^c^/<.001^d^

There were significantly fewer (p < .001) chlamydia and gonorrhea tests performed between April and August 2020 compared to both comparator timeframes (Table 1).

There were also significantly fewer screening tests completed. In addition, significantly fewer (p < .001) unique patients received testing and screening from April to August 2020 compared to earlier timeframes. However, the proportion of tests that met our definition for screening was 70% in April to August 2020 versus 67% in October 2019 to February 2020 versus 64% in April to August 2019. This was the opposite of what we had hypothesized, as we expected the proportion to be lowest from April to August 2020. Even more surprisingly, the proportion of tests meeting our definition of screening was actually significantly higher from April to August 2020 compared to April to August 2019 (p < .001) and October 2019 to February 2020 (p < .01).

## Discussion

Similar to prior studies, we found that overall STI testing decreased for adolescent females during the early phase of the COVID-19 pandemic compared to pre-pandemic levels [3-6]. However, in contrast to our hypothesis that screening would have made up a smaller proportion of STI testing due to the pandemic restrictions, screening tests actually made up a larger proportion of the testing done during the early pandemic compared to earlier time periods. Hence, while other data suggested that screening decreased more than symptomatic testing during the early COVID-19 pandemic due to increased positive test rates, our data do not support this [4-6].

We had hypothesized that screening would make up a smaller proportion of STI testing during the early COVID-19 pandemic because studies have found that routine pediatric care was greatly impacted by the early COVID-19 pandemic, including fundamental care like childhood vaccines [9]. Other studies show that many adolescents and young adults delayed routine care during the summer of 2020 [3-4,10]. In our study, the proportions of tests billed as screening tests were similar across the time periods we assessed. Therefore, the decrease in testing was not the result of a lack of screening exclusively, but a general decrease in both symptomatic testing and screening.

One possible explanation for this finding is that some adolescents sought screening in other venues. While clinics were closed or curtailed during the early pandemic, emergency departments and urgent care centers remained open. So those who wanted testing, even while asymptomatic, could have sought testing in those locations; thus, the proportion of tests done as screening remained steady. Other studies, done prior to the COVID-19 pandemic, found high rates of positive chlamydia and gonorrhea when screening asymptomatic adolescents who presented to the emergency department, suggesting that adolescents who utilize the emergency department for screening may have a higher risk of contracting STIs than those who complete screening during clinic visits [11].

Another possible explanation is that outpatient providers were able to establish strategies to provide laboratory testing despite restrictions, allowing those who did present to care to get screened. One clinic screened patients due for STI screening for COVID-19 symptoms prior to patients presenting for labs in order to maintain access to testing while minimizing COVID-19 exposures [12]. Studies have also shown a spike in interest in and use of home-based testing during the pandemic [12-14]. These and other adjustments may have mitigated the drop in screening brought on by the COVID-19 pandemic. Work is needed to understand the relative contribution of these mitigating strategies in order to develop more flexible responses during future disruptive events, as has been called for by others [5,15].

Lastly, our findings may be reflective of patient behavior. STI testing among adolescents and young adults has been associated with sexual behaviors, such as number of sexual partners and relationship characteristics, in addition to presence of symptoms [16-17]. The impact of social restrictions during the early pandemic period on sexual behavior is unclear [18-19]. If adolescents were engaged in less sexual contact during the early pandemic, they may have sought less testing generally, but would also have been less likely to experience symptoms.

It is important to note that we made a priori assumptions about which billing codes represented asymptomatic screening and which represented symptomatic testing, which may not have been correct. This potential error is a limitation of the study. The analysis was limited by lack of access to testing that was not billed to insurance. In an adolescent population, testing may be done without using patient’s insurance to protect patient confidentiality, such as testing done at Planned Parenthood or local health departments. However, similar numbers of patients were covered over the time periods of interest, so this missing testing is not likely to contribute a substantial bias.

## Conclusions

In summary, we found that the drop in STI testing noted during the early COVID-19 pandemic was not exclusively the result of decreases in asymptomatic screening, as the proportion of tests billed as screening was similar both during and before the early pandemic. The causes of this finding are unclear. Possible causes include changes in adolescent sexual behavior during lockdown and adaptations made by health systems to attempt to maintain access to testing. Future research should explore the impact of adaptive measures taken by health systems to develop strategies for future disruptive events. A more detailed understanding of the impact of pandemic restrictions on adolescent sexual behavior is also needed.
